# Use of Extracorporeal Membrane Oxygenation in Postpartum Management of a Patient with Pulmonary Arterial Hypertension

**DOI:** 10.1155/2018/7031731

**Published:** 2018-01-15

**Authors:** Humna Abid Memon, Zeenat Safdar, Ahmad Goodarzi

**Affiliations:** ^1^Department of Internal Medicine, Houston Methodist Hospital, Houston, TX, USA; ^2^Houston Methodist Pulmonary Hypertension Center, Weill Cornell College of Medicine, Houston, TX, USA; ^3^Houston Methodist Lung Transplant Center, Houston Methodist Hospital, Houston, TX, USA

## Abstract

Current guidelines do not recommend pregnancy in patients with pulmonary arterial hypertension (PAH). This is due to the associated high mortality, which both dissuades PAH patients from becoming pregnant and encourages termination of pregnancy due to high maternal mortality risk. As a result, there is a lack of data and, consequently, there are only general guidelines available for management of pregnancy in PAH patients. Additionally, novel therapeutic strategies such as extracorporeal membrane oxygenation (ECMO), although used in the management of nonpregnant PAH patients as a bridge to lung transplantation, have not been used to treat cardiopulmonary collapse in pregnant PAH patients. In an attempt to bridge this paucity of data, we report the successful use of ECMO in resuscitation and management of a pregnant PAH patient who experienced cardiopulmonary collapse following a caesarian section.

## 1. Introduction

Pregnancy in pulmonary arterial hypertension (PAH) patients predictably induces a state of physiologic stress, compounded by increased intravascular volume, that cannot be accommodated by remodeled pulmonary arteries in PAH [1]. Thus, the puerperal state in patients with PAH can precipitate right heart failure (RHF) and hemodynamic collapse. Given this high risk, these patients are counseled against pregnancy [2] However, some women may still choose to become pregnant. Emerging modalities such as extracorporeal membrane oxygenation (ECMO), in the management of pregnant patients with PAH, have not been explored. Herein, we report the novel use of ECMO in the postpartum management of a PAH patient whose pregnancy was complicated by worsening pulmonary arterial hypertension (PAH) and post-caesarian section cardiopulmonary collapse.

## 2. Case Description

A 20-year-old female, with a known diagnosis of PAH and von Willebrand Disease (vWD), presented to the clinic at 13 weeks of gestation. She was diagnosed with PAH at the age of 5 and had undergone blade followed by balloon atrial septostomy and then subcutaneous (SC) treprostinil was started. On repeat heart catheterization at 14 years of age, tadalafil and bosentan were added to her regimen. At 16 years of age, her care was transitioned to an adult Pulmonary Hypertension (PH) center. An echocardiogram (ECHO) done at that time demonstrated a right ventricular systolic pressure (RVSP) of 88 mm Hg. Bosentan was changed to macitentan due to noncompliance with monthly liver function testing. She was continued on SC infusion of treprostinil at a dose of 38 ng/kg/min, tadalafil 40 mg daily, macitentan 10 mg daily, spironolactone 25 mg daily, digoxin 125 mcg daily, and warfarin.

Despite physicians' recommendations and multiple counseling sessions, the patient became pregnant, citing religious reasons for this decision. On affirmation of her desire to continue her pregnancy, all teratogenic medications such as spironolactone, digoxin, warfarin, and macitentan were stopped while tadalafil and treprostinil were continued. In addition, she was admitted and transitioned from SC treprostinil to intravenous epoprostenol. She did well on this regimen until 25 weeks of gestation when she presented to the emergency department with complaints of recurrent hemoptysis and acute respiratory distress. She had a respiratory rate of 25/min, blood pressure (BP) of 110/65 mm Hg, and oxygen saturation of 86% in room air. While chest examination was unremarkable, with equal and no additional breath sounds bilaterally, a systolic grade 2/6 murmur was auscultated at the left sternal border and a jugular venous distension of 10 cm was noted on her cardiovascular exam.

**Figure 1:**
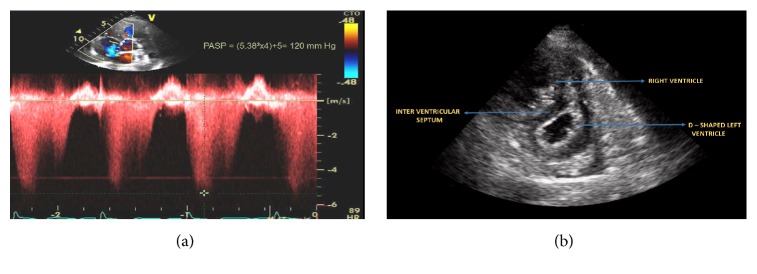
(a) Tricuspid regurgitation signal on echocardiogram at 25 weeks of gestation showing peak velocity of 5.38 m/s and Pulmonary Artery Systolic Pressure (PASP) of 120 mm Hg. (b) Echocardiogram at 25 weeks of gestation showing enlarged right ventricle and D-shaped left ventricle.

The patient was supported with 10 liters (L) of nasal cannula oxygen and an arterial blood gas was obtained, which showed a pH of 7.47, pCO_2_ of 27 mm Hg, and pO_2_ of 147 mm Hg. Additional laboratory tests, consistent with her history of vWD, revealed thrombocytopenia with a platelet count of 75 *κ*/*μ*L. She was hence admitted to the intensive care unit (ICU) for close monitoring. Following admission, her ECHO showed a RVSP that had increased from 80 mm Hg, noted on her ECHO at 13 weeks of gestation, to 120 mm Hg (Figures 1(a) and 1(b)). In light of these findings and her presentation with acute decompensated PAH, a maternal-fetal specialist team was consulted and she was placed on continuous electronic fetal monitoring. Concurrently, betamethasone and magnesium sulfate were administered for expedition of fetal development and seizure prophylaxis, respectively. In addition, a team of specialists, including neonatal intensivist and cardiovascular surgeon, were assembled.

It was on continuous fetal monitoring at 26 weeks of gestation that fetal bradycardia was discovered, with acceleration and moderate variability between 100 and 110 beats/minute, but no decelerations. Due to concern of further decompensation and imminent danger to the patient and the fetus, an emergent caesarian section was undertaken.

Due to significant thrombocytopenia, the procedure was undertaken under general anesthesia and a healthy male infant was successfully delivered. However, during the closure of the hysterotomy, the patient developed ventricular tachycardia and went into cardiac arrest. Following 15 minutes of successful cardiopulmonary resuscitation (CPR), she was placed on mechanical ventilation. Due to precipitation of RHF, the patient was placed emergently on femoral-femoral venoarterial (VA) ECMO for life support. Using the Seldinger technique, a 17-French arterial cannula and a 21-French multistage venous cannula were inserted, and full ECMO support at a flow rate of 3.79 L/min was initiated, in addition to multiple vasopressors, including 5 *μ*g/kg/min of dobutamine, 0.04 units/min of vasopressin, and 9 *μ*g/kg/min of norepinephrine and milrinone. A Swan Ganz catheter was simultaneously placed at the time of ECMO insertion. Her hemodynamics showed a PAP of 139/85 mm Hg, central venous pressure of 11 mm Hg, pulmonary artery wedge pressure of 10 mm Hg, and cardiac index (CI) of 2.1 L/min/m^2^. The patient had a prolonged stay in the ICU and required continuous inotropes and chronotropes support. Her hospital course was further complicated by recurrent pericardial effusions and consequent cardiac tamponade, for which a pericardial window was undertaken along with hemoptysis that required bronchoscopy and cauterization.

**Figure 2:**
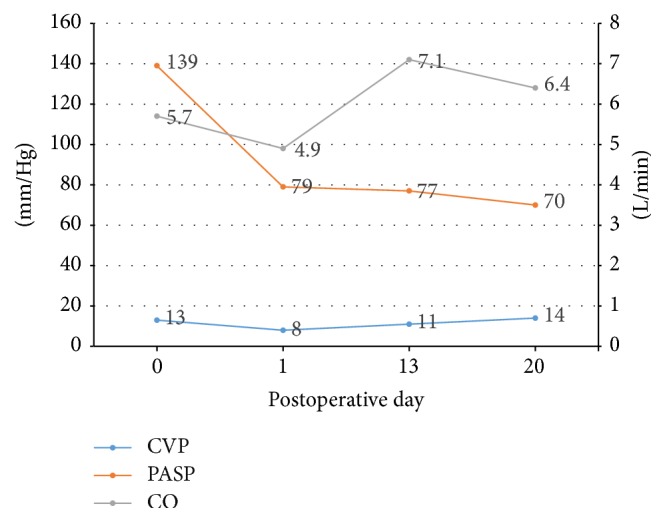
Graph representing changes in pulmonary artery systolic pressure, cardiac output, and central venous pressure after initiation of extracorporeal membrane oxygenation. CVP, central venous pressure; PASP, pulmonary artery systolic pressure; CO, cardiac output.

The use of ECMO and reinitiation of PAH specific therapy slowly improved the patient's hemodynamic parameters (Figure 2 and Table 1). PAH specific therapy initially was limited to intravenous epoprostenol, but, upon normalization of LFTs, macitentan was initiated and as PA pressure gradually trended down to 80 mmHg and her blood pressure improved, sildenafil was added. Her requirement for vasopressors declined and she was gradually weaned off. Simultaneously, ECMO flow rates and support through VA ECMO continued to decrease and she was finally removed from ECMO support on postoperative day (POD) 24. With better control of PA pressures along with aggressive diuresis, ventilation improved and her fraction of inspired oxygen (FiO_2_) requirements declined. However, due to prolonged intubation and unsuccessful weaning from mechanical ventilation, a tracheostomy was performed on POD 30. A subsequent ECHO demonstrated a RVSP of 69 mm Hg, cardiac output of 4.3 L/min, and CI of 2.5 L/min/m^2^. After switching back to SC treprostinil, she was discharged from the hospital on POD 101, on the following regimen: ambrisentan 10 mg daily, SC treprostinil 30 ng/kg/min, sildenafil 20 mg three times daily, spironolactone 50 mg daily, and digoxin 62.5 mcg daily.

## 3. Discussion

PAH is defined as resting mPAP ≥ 25 mmHg with pulmonary artery wedge pressure (PCWP) ≤ 15 mm Hg and pulmonary vasculature resistance (PVR) > 3 Woods units [3–5]. Several physiologic changes occur during pregnancy, including an increase in intravascular plasma volume, stroke volume, and cardiac output [1]. Normal pulmonary vasculature responds to these changes to maintain a normal mPAP by decreasing PVR through vasodilation and recruiting previously nonperfused pulmonary vessels [6, 7]. However, in pregnant women with PAH, the pulmonary vasculature lacks the ability to mount this response due to increased endothelin activation along with nitric oxide and prostacyclin deficiency [6]. This places added stress on the already compromised cardiopulmonary system in PAH. The resultant right ventricular failure and cardiovascular collapse are the primary causes of high mortality in gravid patients with PAH [8].

With the use of PAH specific therapy, mortality has decreased in nongravid as well as pregnant females with PAH [7]. Current data suggests that mortality rates in pregnant women with PAH have declined from 50% to 17–33% [7, 9–14]. Despite this decrease, mortality in these patients still remains high, with the highest rates occurring postpartum [15]. Use of ECMO as a bridge to transplantation or recovery in patients with decompensated PAH has been reported in several cases [16–18]. In these patients, not only do targeted drug therapies prove to be inadequate, but their systemic vasodilator effect can potentially compound the worsening right ventricular failure and precipitate impending cardiogenic shock. Although limited, the use of extracorporeal life support in pregnancy and postpartum patients with cardiorespiratory failure has also been on the rise in the last five years [19, 20]. In reviewed cases, the overall maternal and fetal mortality was noted to be around 70% and 80%, respectively [21]. Despite this, there are no designated indications for the use of ECMO in pregnant or postpartum patients with PAH, which has been limited to managing cardiorespiratory collapse arising as a complication of various pathologies, not inclusive of PAH [19–21].

Hence, we report a novel method of managing PAH in the postpartum state. As demonstrated by our case, use of ECMO can help stabilize postpartum patients with PAH during a time when the PA pressures are at their peak and maternal mortality is at its highest. We believe that this can play an integral role in successfully managing the complicated hemodynamics in pregnant or postpartum patients with PAH.

Currently, recommendations for management of PAH in pregnancy and the postpartum state include monitoring by a multidisciplinary team, including PAH expert, high risk obstetrician, cardiothoracic surgeon, and anesthesiologist, along with early initiation of PAH specific therapy and close monitoring of fetus [7, 13]. Notably, while our patient had a favorable outcome, pregnancy in PAH patients carries a high risk to both mother and fetus. This holds true especially if management of pregnancy at an experienced PH center is not possible or accessible. Hence, per current guidelines, at time of diagnosis, PAH patients should be strongly dissuaded from becoming pregnant and be concurrently counseled regarding effective contraceptive methods. ECMO as an ancillary tool has not been explored in the management of the accompanying RHF and cardiopulmonary collapse in these high risk PAH patients. The unique risks associated with ECMO in the partum, peripartum, and postpartum period are not yet known, with concerns of both hypercoagulability and hemorrhage. However, as demonstrated by this case, the use of ECMO in pregnant PAH patients warrants further exploration.

## Figures and Tables

**Table 1:** Hemodynamic measurements obtained after cardiac arrest and insertion of extracorporeal membrane oxygenation.

Swan Ganz parameters	POD 0	POD 1	POD 13	POD 20
CVP (mm Hg)	13	8	11	14
PASP (mm Hg)	139	110	114	89
PADP (mm Hg)	85	60	59	41
mPAP (mm Hg)	103	79	77	60
CO (l/min)	---	4.9	7.1	6.4
CI (l/m/m2)	----	3.2	4.5	4.1

CVP = central venous pressure, PASP = pulmonary artery systolic pressure, PADP = pulmonary artery diastolic pressure, mPAP = mean pulmonary artery pressure, CO = cardiac output, CI = cardiac index, SVR = systemic venous resistance, and SvO_2_ = systemic venous oxygen saturation.

## References

[1] Madden B. P. (2009). Pulmonary hypertension and pregnancy. *International Journal of Obstetric Anesthesia*.

[2] Galiè N., Humbert M., Vachiery J. (2015). 2015 ESC/ERS Guidelines for the Diagnosis and Treatment of Pulmonary Hypertension: The Joint Task Force for the Diagnosis and Treatment of Pulmonary Hypertension of the European Society of Cardiology (ESC) and the European Respiratory Society (ERS): Endorsed By: Association for European Paediatric and Congenital Cardiology (AEPC), International Society for Heart and Lung Transplantation (ISHLT). *European Respiratory Journal*.

[3] Hoeper M. M., Bogaard H. J., Condliffe R. (2013). Definitions and diagnosis of pulmonary hypertension. *Journal of the American College of Cardiology*.

[4] McLaughlin V. V., Archer S. L., Badesch D. B. (2009). ACCF/AHA 2009 expert consensus document on pulmonary hypertension: a report of the American College of Cardiology Foundation Task Force on Expert Consensus Documents and the American Heart Association: developed in collaboration with the American College of Chest Physicians, American Thoracic Society, Inc., and the Pulmonary Hypertension Association. *Circulation*.

[5] Galiè N., Hoeper M. M., Humbert M. (2009). Guidelines for the diagnosis and treatment of pulmonary hypertension: The Task Force for the Diagnosis and Treatment of Pulmonary Hypertension of the European Society of Cardiology (ESC) and the European Respiratory Society (ERS), endorsed by the International Society of Heart and Lung Transplantation (ISHLT). *European Heart Journal*.

[6] Safdar Z. (2013). Pulmonary arterial hypertension in pregnant women. *Therapeutic Advances in Respiratory Disease*.

[7] Hemnes A. R., Kiely D. G., Cockrill B. A. (2015). Statement on pregnancy in pulmonary hypertension from the pulmonary vascular research institute. *Pulmonary Circulation*.

[8] Gei A., Montufar-Rueda C. (2014). Pulmonary hypertension and pregnancy: An overview. *Clinical Obstetrics and Gynecology*.

[9] Weiss B. M., Zemp L., Seifert B., Hess O. M. (1998). Outcome of pulmonary vascular disease in pregnancy: A systematic overview from 1978 through 1996. *Journal of the American College of Cardiology*.

[10] Bédard E., Dimopoulos K., Gatzoulis M. A. (2009). Has there been any progress made on pregnancy outcomes among women with pulmonary arterial hypertension?. *European Heart Journal*.

[11] Kiely D. G., Condliffe R., Webster V. (2010). Improved survival in pregnancy and pulmonary hypertension using a multiprofessional approach. *BJOG: An International Journal of Obstetrics & Gynaecology*.

[12] Jaïs X., Olsson K. M., Barbera J. A. (2012). Pregnancy outcomes in pulmonary arterial hypertension in the modern management era. *European Respiratory Journal*.

[13] Duarte A. G., Thomas S., Safdar Z. (2013). Management of pulmonary arterial hypertension during pregnancy: A retrospective, multicenter experience. *CHEST*.

[14] Katsuragi S., Yamanaka K., Neki R. (2012). Maternal outcome in pregnancy complicated with pulmonary arterial hypertension. *Circulation Journal*.

[15] Shaun Smith J., Mueller J., Daniels C. J. (2012). Pulmonary arterial hypertension in the setting of pregnancy: A case series and standard treatment approach. *Lung*.

[16] Rosenzweig E. B., Brodie D., Abrams D. C., Agerstrand C. L., Bacchetta M. (2014). Extracorporeal membrane oxygenation as a novel bridging strategy for acute right heart failure in group 1 pulmonary arterial hypertension. *ASAIO Journal*.

[17] Abrams D. C., Brodie D., Rosenzweig E. B., Burkart K. M., Agerstrand C. L., Bacchetta M. D. (2013). Upper-body extracorporeal membrane oxygenation as a strategy in decompensated pulmonary arterial hypertension. *Pulmonary Circulation*.

[18] Tsai M.-T., Hsu C.-H., Luo C.-Y., Hu Y.-N., Roan J.-N. (2015). Bridge-to-recovery strategy using extracorporeal membrane oxygenation for critical pulmonary hypertension complicated with cardiogenic shock. *Interactive CardioVascular and Thoracic Surgery*.

[19] Agerstrand C., Abrams D., Biscotti M. (2016). Extracorporeal membrane oxygenation for cardiopulmonary failure during pregnancy and postpartum. *The Annals of Thoracic Surgery*.

[20] Moore S. A., Dietl C. A., Coleman D. M. (2016). Extracorporeal life support during pregnancy. *The Journal of Thoracic and Cardiovascular Surgery*.

[21] Sharma N. S., Wille K. M., Bellot S. C., Diaz-Guzman E. (2015). Modern use of extracorporeal life support in pregnancy and postpartum. *ASAIO Journal*.

